# State biomarkers for Machado Joseph disease: Validation, feasibility and responsiveness to change

**DOI:** 10.1590/1678-4685-GMB-2018-0103

**Published:** 2019-06-10

**Authors:** Gabriel Vasata Furtado, Camila Maria de Oliveira, Gabriela Bolzan, Jonas Alex Morales Saute, Maria Luiza Saraiva-Pereira, Laura Bannach Jardim

**Affiliations:** 1 Programa de Pós-Graduação em Genética e Biologia Molecular, Universidade Federal do Rio Grande do Sul (UFRGS), Porto Alegre, RS, Brazil; 2 Laboratório de Identificação Genética, Hospital de Clínicas (HCPA), Porto Alegre, RS, Brazil; 3 Serviço de Genética Médica, Hospital de Clínicas de Porto Alegre (HCPA), Porto Alegre, RS, Brazil; 4 Faculdade de Medicina, Universidade Federal do Rio Grande do Sul (UFRGS), Porto Alegre, RS, Brazil; 5 Programa de Pós-Graduação em Medicina: Ciências Médicas, Universidade Federal do Rio Grande do Sul (UFRGS), Porto Alegre, RS, Brazil; 6 Departamento de Bioquímica, Universidade Federal do Rio Grande do Sul (UFRGS), Porto Alegre, RS, Brazil; 7 Departamento de Medicina Interna, Universidade Federal do Rio Grande do Sul (UFRGS), Porto Alegre, RS, Brazil

**Keywords:** Biomarkers, neurophysiology, Machado-Joseph disease, spinocerebellar ataxia type 3

## Abstract

Machado-Joseph disease (SCA3/MJD) is the most common spinocerebellar ataxia worldwide, and particularly so in Southern Brazil. Due to an expanded polyglutamine at ataxin-3, SCA3/MJD presents a relentless course with no current disease modifying treatment. Clinical scales used to measure SCA3/MJD progression present moderate effect sizes, a major drawback for their use as main outcomes in clinical trials, given the rarity and slow progression of the disease. This limitation might be overcome by finding good surrogate markers. We present here a review of studies on peripheral and neurophysiological markers in SCA3/MJD that can be candidates for state biomarkers. Data on markers already studied were summarized, giving emphasis on validation against clinical scale, and responsiveness to change. While some biological fluid compounds and neurophysiological parameters showed poor responsiveness, others seemed to be good candidates. Some potential candidates that are waiting for responsiveness studies were serum levels of neuron specific enolase, vestibulo-ocular reflex and video-oculography. Candidates evaluated by RNA and microRNA expression levels need further studies to improve their measurements. Data on peripheral levels of Beclin-1 and DNAJB1 are promising but still incipient. We conclude that several potential candidates should follow onto validating studies for surrogate state biomarkers of SCA3/MJD.

## Introduction

Machado-Joseph disease, also known as spinocerebellar ataxia type 3 (SCA3/MJD), is an autosomal dominant spinocerebellar ataxia caused by an expanded CAG repeat (longer than 51 triplets) at *ATXN3* gene, giving rise to an expanded polyglutamine (polyQ) at ataxin-3 protein (Saute and Jardim, 2015). With a mean age at onset of 34-40 yo (Dürr *et al.*, 1996; Schöls *et al.*, 1997; Tang *et al.*, 2000; Globas *et al.*, 2008; de Castilhos *et al.*, 2014; du Montcel *et al.*, 2014; Zhou *et al.*, 2014), SCA3/MJD involves predominantly the cerebellar, pyramidal, extrapyramidal, motor neuron, and oculomotor systems. Gait ataxia is commonly the first symptom, followed by diplopia, dysarthria, spasticity, dystonic movements, sensory losses and other findings, in different combinations (Jardim *et al.*, 2001; Saute and Jardim, 2015). SCA3/MJD is very heterogeneous and never exclusively ataxic. Currently there is no disease modifying treatment and SCA3/MJD presents a relentless progression, with an average survival of 21.18 years after onset of symptoms (Kieling *et al.*, 2007). However, several lines of pre-clinical research gave rise to good candidate treatments targeting different cellular and molecular pathways, a scenario in which robust designs of clinical trials will be paramount for the success of the therapeutic endeavor (Li *et al.*, 2015; Duarte-Silva *et al.*, 2018; Matos *et al.*, 2018). Considering the very slow progression of SCA3/MJD on clinical scales and the rarity of the disease, state biomarkers might be important surrogate endpoints for these future clinical studies.

Biomarkers are substances, structures, or processes that can be measured in the body or its products and influence or predict the incidence or outcome of disease, of treatments, or of environmental exposures" (WHO International Programme on Chemical Safety, 2001). Trait biomarkers are present prior to start of the disease process, while state biomarkers are due to disease process or due to a therapy response, and mirror disease progression. State biomarkers should be correlated to clinically meaningful endpoints. If state biomarkers show advantages when comparing to clinical endpoints, they can replace them in clinical trials (Aronson, 2005). This is the case of a biomarker whose changes can be measured easily and in a more sensitive way than clinical endpoints. Such surrogate markers are especially important for phase II, randomized clinical trials (phase II RCT) addressed to raise preliminary evidence of efficacy for a given drug, especially in the context of rare diseases.

Efficacy of a given treatment is most fully demonstrated when outcomes of treated versus control groups vary according to a minimal clinically important difference (MCID); and MCID were never clearly determined to SCA3/MJD. The closest to that was obtained by the Scale of Assessment and Rating of Ataxia (SARA), a validated semi-quantitative scale that progresses between 0.65 and 1.56/40 points per year (Schmitz-Hübsch *et al.*, 2006, 2010; Chan *et al.*, 2011; Ashizawa *et al.*, 2013; Jacobi *et al.*, 2015), and where 1.5 points were noted by patients according to the patients global impression of improvement (PGI-I). Nevertheless, disease progression is slow as measured by SARA and by all other clinical scales in use - the International Cooperative Ataxia Rating Scale (ICARS) (Trouillas *et al.*, 1997), Neurological Examination Score for Spinocerebellar Ataxias (NESSCA) (Kieling *et al.*, 2008), Composite-Cerebellar-Functional-Score (CCFS) (du Montcel *et al.*, 2008), and the Inventory of Non-Ataxia Symptoms (INAS) (Schmitz-Hübsch *et al.*, 2008). Clinical trials should be tailored to face this issue.

A drawback shared by all clinical scales is their large variability, which can reduces their effect sizes (ES), either by the Cohens effect size (CES), or the standardized response mean (SRM) (Streiner and Norman, 2008; Saute *et al.*, 2012). The average SRM obtained for SARA scale was 0.5 (Schmitz-Hübsch *et al.*, 2010). Considering SARA SRM with a progression of 1 point per year, between 175 and 328 subjects would be needed in each arm to show a 50% reduction in the disease progression rate in a future trial (Schmitz-Hübsch *et al.*, 2010; Chan *et al.*, 2011; Saute *et al.*, 2015). For a rare disease, these numbers are generally unfeasible. This might be overcome by the discovery of a good surrogate, or a set of surrogate markers, with ES larger than those presented by current clinical scales.

Since biomarkers are much needed, we aimed to review the state of art of potential surrogate markers of disease state in SCA3/MJD, focusing on neurophysiology markers and biological fluid compounds. Candidates for state biomarkers were included, provided that some preliminary evidence in humans was already published. Validation against a meaningful clinical endpoint, feasibility, rate of change in time (progression rate), and responsiveness to change were the parameters in focus.

## Materials and Methods

### Search methods

We performed a search in MEDLINE up to November, 2017. The search terms were (Machado-Joseph disease OR spinocerebellar ataxia) AND (Biomarker* OR Biologic* Marker* OR Laboratory Marker* OR Serum Marker* OR Surrogate Endpoint* OR Biochemical Marker* OR Immune Marker* OR immunologic* marker* OR miRNA) OR (Biomarker* OR Electroencephalography* OR Evoked potentials* OR Transcranial Magnetic Stimulation* OR Quantitative Motor Features* OR Vestibular* OR Video-Oculography* OR Nerve Conduction Studies* OR Electromyography*).

In addition, a manual search for references known by authors that were not covered by the above search strategy was also performed, and such studies were included.

### Criteria for including studies

We included studies describing biological fluid compounds and neurophysiological measures that could be candidate for state biomarkers. Case-control and prospective studies and clinical trials were also included, provided that quantitative information on their candidate markers were given.

Original studies on cellular or animal models, as well as studies in humans lacking quantitative data, or when specific SCA3/MJD diagnosis was missing, case reports, case series (without controls), reviews, comments, editorials, and guidelines, and studies written in languages other than English were excluded. Neuroimaging studies were addressed in a recent systematic review (Klaes *et al.*, 2016), and therefore were not included in this review.

Clinical rating scales or scores for cerebellar ataxia and studies whose design was intended to identify a trait biomarker - for instance, studies searching for modifiers of age at onset - were not within the scope of this review.

### Study organization

Results were presented in two groups of candidate biomarkers: biological fluid compounds and neurophysiology characteristics. The main scientific queries were related to evidences on validation against a clinical scale, responsiveness, and clinical significance. If already estimated, sample sizes for future trials were mentioned as well.

### Sensitivity to change

Cohen’s Effect Size (CES) or the Standardized Response Mean (SRM) were provided to candidate biomarkers, when available. The following formulas were applied: (1) mean score change/standard deviation (SD) of score at baseline (for CES), and (2) mean score change/SD of score change (for SRM) when data were available and CES, or SRM were not determined.

## Results

### Biological fluid compounds

**Figure 1:**
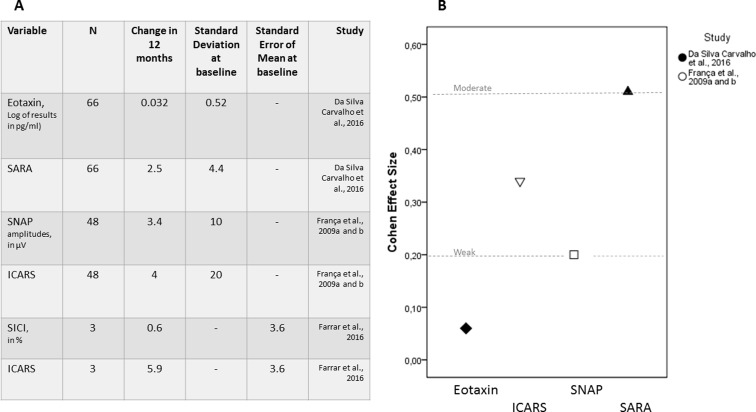
Candidate biomarkers that have been followed longitudinally in SCA3/MJD subjects. (A) Summary of the longitudinal data obtained for eotaxin and Scale for Assessment and Rating of Ataxia (SARA); sensory nerve action potential (SNAP) amplitudes of sural nerves and International Cooperative Ataxia Rating Scale (ICARS); and short-interval intracortical inhibition (SICI) of motor evoked potentials and ICARS. (B) Cohen effect sizes, when available or when estimation was possible.

Table 1 summarizes data on biological fluid compounds reported on SCA3/MJD and included in the present review. Studies with positive results related to disease state, on neurotrophic/growth factors, inflammatory mediators, and astrocyte activators, markers of neuronal and glial loss, oxidative stress, and protein quality control systems markers are described below. Longitudinal data was available only for eotaxin levels, and the effect size of this candidate is described in Figure 1.

**Table 1:** Peripheral compounds studied in spinocerebellar ataxia type 3/Machado-Joseph disease (SCA3/MJD) carriers, and prone to be candidates for state biomarkers of this disease. Compounds are presented according to area of metabolism.

Candidate Marker	Reference	Sample Size		Sample	Comparison with controls		Correlations were found among SCA3/MJD subjects?	
		SCA3/MJD Cases	Controls		SCA3/MJD Cases	Controls	With clinical scales	With disease duration
**Neurotrophic/Growth factors**								
Insulin	Saute *et al.*, 2011	46	42	Serum	Insulin levels: 6.2(3.5) uIU/mL*	Insulin levels: 9.5(6) uIU/mL	No	No
				Serum	HOMA2-%B: 83.9(35)	HOMA2-%B: 92.9(50.5)	No	No
				Serum	Log(HOMA2-%S): 4.8(0.55)**	Log(HOMA2-%S): 4.35(0.63)	No	No
IGF-1	Saute *et al*., 2011	46	42	Serum	Total IGF-1: 114.5(32.2) ng/mL	Total IGF-1: 117.4(36.3) ng/mL	No	No
				Serum	Free IGF-1 (IGF-1/IGFBP-3 molar ratio): 0.36(0.24)*	0.23(0.12)	No	No
IGFBP-1	Saute *et al.*, 2011	46	42	Serum	2.67(1.8) ng/mL **	1.32(0.98)	No	No
IGFBP-3	Saute *et al.*, 2011	46	42	Serum	1.4(0.8) ug/mL**	2.01(0.36)	No	No
**Activation of pro-inflammatory factors**								
*FCGR3B* gene	Raposo *et al.*, 2015	12 (DC) 42 (CC)	12 (DC) 35 (CC)	RNA from peripheral blood	FC: 2.597*; SD not informed	NA	ND	Yes* FC and SD not informed
*TNFSF14* gene	Raposo *et al.*, 2015	12 (DC) 42 (CC)	12 (DC) 35 (CC)	RNA from peripheral blood	FC: 1.687; SD not informed	NA	ND	Yes (short disease duration only)* FC and SEM not informed
*SELPLG* gene	Raposo *et al.*, 2015	12 (DC) 42 (CC)	12 (DC) 35 (CC)	RNA from peripheral blood	FC: 1.324*; SD not informed	NA	ND	No
**Activation of astrocytes**								
miR-34b	Shi *et al*., 2014	9 (DC) 35 (VC)	7 (DC) 25 (VC)	Serum	Up-regulated: Ratio Cases/Controls: 4.79*** SD not informed	NA	No	No
miR-29a	Shi *et al*., 2014	9 (DC) 35 (VC)	7 (DC) 25 (VC)	Serum	Down-regulated: Ratio Controls/Cases: 4.7*	NA	No	No
miR-25	Shi *et al*., 2014	9 (DC) 35 (VC)	7 (DC) 25 (VC)	Serum	Down-regulated: Ratio Controls/Cases: 2.04*	NA	No	Yes (longer disease duration only)* atio and SEM not informed
miR-125b	Shi *et al*., 2014	9 (DC) 35 (VC)	7 (DC) 25 (VC)	Serum	Down-regulated: Ratio Controls/Cases: 2.1*	NA	No	Yes (longer disease duration only)* Ratio and SEM not informed
GFAP	Shi *et al*., 2014	136	151	Serum	8.86(4.33) ng/mL **	3.93 2.38	No	No
Eotaxin	da Silva Carvalho *et al.,*2016 Saute *et al.*, 2014	66 (Symptomatic) 13 (Asymptomatic)	43	Serum	Symptomatic carriers logEotaxin: 1.3 (SE=0.1) (SD: 0.50724). Asymptomatic carriers logEotaxin: 2.3 (SE=0.2) ***	Controls: 1.33 (SE=0.09)	No	No
**G-protein coupled receptors**								
*P2RY13* gene	Raposo *et al.*, 2015	12 (DC) 42 (CC)	12 (DC) 35 (CC)	RNA from peripheral blood	FC: 1.665*; SD not informed	NA	ND	No
**Enzyme**								
*CLC* gene	Raposo *et al.,*2015	12 (DC) 42 (CC)	12 (DC) 35 (CC)	RNA from peripheral blood	FC: 2.041 SD not informed	NA	ND	Yes* FC and SD were not informed
**Others**								
*SLA* gene	Raposo *et al.*, 2015	12 (DC) 42 (CC)	12 (DC)35 (CC)	RNA from peripheral blood	FC: 1.333 SD not informed;	NA	ND	Yes (short disease duration only)* FC and SD not informed
**Markers of neuronal/glial loss**								
NSE	Tort *et al.*, 2005	22	22	Serum	8.05(4.2) ng/mL ***	4.65 (1.80) ng/mL	EDSS (R=-0.729*)	No
	Zhou *et al.*, 2011	102	100	Serum	6.95(2.83) ng/mL***	4.83 (1.70) ng/mL	ICARS R=0.242* SARA R=0.248* ICARS = 26.68 (13.37)SARA = 9.98 (4.65)	R=0.259**
S100B	Tort *et al.*, 2005	22	22	Serum	0.108(0.073) ug/l	0.082 (0.042) ug/l	No	R=0.452*
	Zhou *et al.*, 2011	102	100	Serum	0.07(0.06) ng/ml ***	0.05 (0.02) ng/ml	No	No
Neurofilament	Wilke *et al.,*2018	8	16	Serum	70 pg/ml (range: 40 to 105) ***	22 pg/ml (8 to 35)	No	No
**Oxidative Stress Markers**								
DCFH-DA	de Assis *et al.*, 2017	58 (Symptomatic) 12 (Presymptomatic)	47	Serum	Symptomatic SCA3/MJD: 335.7 nmol/mg of protein (SE 21.2)*** Presymptomatic individuals: 91.8 nmol/mg of protein (SE 42.2)	Controls: 182.8 nmol/mg of protein (SE 20.3)	No	No
	Pacheco *et al.,*2013	7	7	Serum	172.126(66.49) nmol/mg of protein	171.606(20.395) nmol/mg of protein	NA	NA
SOD	de Assis *et al.*, 2017	58 (Symptomatic) 12 (Presymptomatic)	47	Serum	Symptomatic: 9.3 (SE 0.5) U/mg of protein * Presymptomatic: 12.3 (SE 1.1) U/mg of protein	Controls: 10.8 (SE 0.5) U/mg of protein	No	No
GSH-Px	de Assis *et al.*, 2017	58 (Symptomatic) 12 (Presymptomatic)	47	Serum	Symptomatic: 56.3 (SE 2.4) U/mg of protein *** Presymptomatic: 76.8 U/mg of protein (SE 5.2)	70.3 (SE 2.3) U/mg of protein	NESSCA R=-0.309* NESSCA = 14.27 (4.7) SE = 0.598	No
Thiol groups	Pacheco *et al.*, 2013	7	7	Serum	0.112 nmol/mL of erythrocytes (0.032)***	0.275 nmol/mL of erythrocytes (0.047)	NA	NA
Catalase	Pacheco *et al.*, 2013	7	7	Serum	40.7(10.1) mol of H_2_O_2_/mL of erythrocytes/min*	27.67(10.01) mol of H_2_O_2_/mL of erythrocytes/min	NA	NA
DNA damage index (comet assay)	Pacheco *et al.*, 2013	7	7	Lymphocytes	Higher level of DNA damage in SCA3/MJD individuals* (raw values were not presented)	Higher level of DNA damage in SCA3/MJD individuals* (raw values were not presented)	NA	NA
Others (total polypheno, protein carbonyl, TBARS)	Pacheco *et al.*, 2013	7	7	Serum/Plasma	Total polyphenols: 0.632 (0.498) mg/mL Protein carbonyl: 2.751 (0.181) nmol/mg protein TBARS: 44.534 (33.01) nmol/mL of erythrocytes	Total polyphenols: 1.029 (0.770) mg/mL Protein carbonyl: 2.665 (0.471) nmol/mg protein TBARS: 31.786 (32.312) nmol/mL of erythrocytes	NA	NA
**Protein quality control systems**								
Beclin-1	Nascimento-Ferreira *et al.*, 2011	2	1	Fibroblast (protein)	Case 1 – 0.86 (0.087) Case 2 – 0.69 (0.05)	1.15 (0.038)	NA	NA
	Onofre *et al.,*2016	5	4	Fibroblast- (protein and mRNA)	Lower Beclin-1 levels in cases .* Raw values were not presented	Lower Beclin-1 levels in cases.* Raw values were not presented	NA	NA
DNAJB1	Zijlstra *et al.*, 2010	22	6	Fibroblast	No. Raw values were not presented	NA	NA	NA
HSPB1	Zijlstra *et al.*, 2010	22	6	Fibroblast	Higher levels in cases*. Raw values were not presented	NA	NA	NA
HSPA1A and HSPA8	Zijlstra *et al.*, 2010	22	6	Fibroblast	No. Raw values were not presented	NA	NA	NA

* *p* < 0.05; ** *p* < 0.01; *** *p* < 0.001

IGF-1, insulin-like growth factor 1; IGFBP, insulin-like growth factor binding protein; GFAP, glial fibrillary acidic protein; NSE, neuron specific enolase; DCFH-DA , 2’,7’-dichlorofluorescein diacetate; SOD, superoxide dismutase; GSH-Px, glutathione peroxidase; TBARS, thiobarbituric acid reactive substances; DC, discovery cohort; CC, confirmation cohort; HOMA, Homeostasis Model Assessment; HOMA2-%B, HOMA2 - steady-state β-cell function; HOMA2-%S, HOMA2 - peripheral insulin sensitivity; EDSS, Expanded Disability Status Scale; ICARS, international cooperative ataxia rating scale; SARA scale for the assessment and rating of ataxia; NESSCA, Neurological Examination Score for Spinocerebellar Ataxias; IQ, interquartile; NA, not available; ND, not done; SD, standard deviation; SE, standard error; FC, fold change; SEM, standard error of mean.

Among compounds associated to symptomatic status of SCA3/MJD carriers, only serum neuron-specific enolase (NSE) levels and glutathione peroxidase activity (GSH-Px) were found to be related to SCA3/MJD by two independent case/control studies each (Tort *et al.*, 2005; Zhou *et al.*, 2011; Pacheco *et al.*, 2013; de Assis *et al.*, 2017). NSE is a peripheral marker of neuronal disruption, and increased levels of this protein are associated to neuronal death. However, inconsistent associations were found between NSE and clinical scales (Table 1). GSH-Px activity reflects antioxidant defense capacity. A moderate inverse correlation of this marker was shown with NESSCA, and differences were observed between symptomatic and presymptomatic phases of the disease (de Assis *et al.*, 2017).

Some biological fluid compounds were associated to SCA3/MJD or to disease severity by single studies using unbiased approaches. Pro-inflammatory factors were particularly prominent among them. After a transcriptome-wide gene expression profile approach, quantitative PCR (qPCR) confirmed upregulation of FCGR3B and SELPLG in SCA3/MJD, and the first one was related to disease duration (Raposo *et al.*, 2015). Another unbiased approach analyzed microRNAs (miRs) of peripheral blood samples. miRs are post-transcriptional repressors that can regulate gene expression at different levels. The expression of four specific miRs was found to be up- or down-regulated in SCA3/MJD patients; some of them being involved in astrocyte proliferation. Of note, a down-regulated expression pattern of miR-25 and miR125b was associated to longer disease duration (Shi *et al.*, 2014). Another unbiased approach evaluated serum cytokines levels and higher levels of serum eotaxin, a cytokine secreted by eosinophils and related to astrocytes in central nervous system (CNS). These were found in asymptomatic carriers when compared to both symptomatic patients and controls. A reduction in the levels of this protein was demonstrated in the symptomatic period a year later (da Silva Carvalho *et al.*, 2016). Eotaxin levels and SARA scores obtained simultaneously in these carriers (Saute *et al.*, 2014) were both broadly dispersed, but the ES of Eotaxin (0.06) was smaller than the ES of SARA (0.50) (Figure 1).

### Neurophysiology

Table 2 summarizes data on neurophysiological candidates found by the present literature review. Longitudinal data was available for one parameter of motor evoked potentials (MEP) and for one parameter of peripheral neurophysiology, but the effect size could be estimated for the latter only (Figure 1).

**Table 2:** Neurophysiological findings obtained in spinocerebellar ataxia type 3/Machado-Joseph disease (SCA3/MJD) carriers, and prone to be candidates for state biomarkers of this disease.

Candidate Marker	Reference	Sample size		Comparison with controls		Correlations were found among SCA3/MJD subjects?	
		SCA3/MJD Cases	Controls	SCA3/MJD Cases	Controls	With clinical scales	With disease duration
**Polysomnography**							
Sleep efficiency (%)	Chi et al., 2013	15	16	68.4 (15.7)**	82.8 (9.3)	ICARS: r = -0.786***	No
REM sleep percentage (%)				6.8 (6.1)***	15.0 (4.9)	ICARS: r = -0.595*	No
**Central neurophysiology**							
Movement-evoked potentials triggered by transcranial magnetic stimulation (MEP): central motor conduction time	Yokota et al., 1998	10	16	4.5(0.8)	4.8(1.1)	ND	No
	Schwenkreis et al., 2002	12	14	6.9 (0.9)	6.6 (1.1)	ND	ND
	Jhunjhunwala et al., 2013	6	32	6.8 (1.5)***	4.8 (0.6)	No	ND
	Farrar et al., 2016	11 (2 pre-ataxic)	62	7.5 (0.4)***	5.3 (0.2)	ICARS: r = 0.81 **	ND
MEP amplitude	Yokota et al., 1998	10	16	0.70 (0.19)**	0.39 (0.13)	ND	No
MEP: resting motor threshold	Schwenkreis et al., 2002	12	14	48.3 (7.6)	49.4 (10.3)	ND	ND
	Jhunjhunwala et al., 2013	6	32	49.8 (8.8)**	41.5 (6.6)	ICARS: No	ND
	Farrar et al., 2016	11 (2 pre-ataxic)	62	62.9 (3.2)	59.5 (1.0)	ND	ND
MEP: intracortical facilitation	Schwenkreis et al., 2002	12	14	101.4 (29.2)***	157.5(26.5)	ND	ND
Threshold tracking paired-pulse transcranial magnetic stimulation : short intracortical inhibition (SICI) (in %)	Farrar et al., 2016	11 (2 pre-ataxic)	62	-1.3 (1.4)***	10.3 (0.7)	ICARS: r = -0.78**	ND
Movement-evoked potentials: late BP with dominant (right) hand movements	Lu et al., 2008	9	8	0.37 (0.75)**	2.40 (1.38)	ND	ND
Suppression of the auditory evoked potential P50 (hippocampus and brainstem)	Ghisolfi et al., 2004	12	24	76.2 (7.3) ***	42.1 (4.4)	ND	No
**Vestibular system**							
Ocular Vestibular Evoked Myogenic Potentials (oVEMP, n10)	Ribeiro et al., 2015	14	20	10.6 (1.4)	10.5 (0.9)	ND	ND
Vestibulo-ocular reflex (VOR) by search coils; gain	Gordon et al., 2014	10	7	0.35 to 0.76 (mean=0.56(15)	0.73 to 0.97	SARA: No	ND
Vestibulo-ocular reflex (VOR) by Video-oculography. Head velocity to eye velocity linear regression (VORr)	Luis et al., 2016	15	40	0.50 (0.30) **	0.94 (0.08)	SARA: r = –0.4**	ND
**Video-oculography**							
Gaze-evoked eye movements (GEEM), horizontal. Frequency (Hz)	Wu et al., 2017	44 symptomatic 12 pre-ataxic	40	1.65(0.75 (symptomatic)*** 0.83 (0.5 (pre-ataxic) **	0.09 (0.15)	SARA: r=0.593**	r=0.550**
Average amplitude of horizontal GEEM				3.40(2.30) *** 1.60(0.66) ***	0.31 (0.55)	SARA: r = 0.760**	r = 0.526**
Horizontal mean pursuit gain (%)				69.4(10.8)*** 81.3 (8.0)	87.9 (4.1)	SARA: r = -0.642**	r = -0.470**
Upward peak saccade velocity (°/seconds)				338(109.3) *** 424(81.6) ***	563 (100.5)	SARA: r = -0.397**	r = -0.282*
Upward saccadic accuracy (%)				85.1(16.0 * 93.0(9.0)	94.4 (7.4)	SARA: r = -0.547**	r = -0.471**
Total antisaccadic error rate (%)				66.8(22.9) *** 36.4(24.1) ***	19.2 (14.0)	SARA: r = 0.330**	r = 0.360**
**Peripheral neurophysiology**							
Compound muscle action potential (CMAP) amplitudes (mV) (tibial)	Klockgether et al., 1999	58	91	16.4 (7.6)	23.0 (6.9)	ND	No
	França et al., 2009a	48	20	9.6 (4.2)	9.0 (1.7)	ND	ND
	Suga et al., 2014	17	80	9.2 (4.3)**	12.6 (3.3)	ND	ND
Sensory nerve action potential (SNAP) amplitudes (μV) (sural)	Klockgether et al., 1999	58	91	6.7 (4.7)#	17.8 (7.5)	ND	No
	França et al., 2009ª França et al., 2009b	48	20	12.1 (9.9)**	24.1 (6.3)	ND	No
	Suga et al., 2014	16	80	11.1 (8.2)**	19.3 (9.7)	ND	ND
Motor nerve conduction velocity, tibial nerve (m/s)	Klockgether et al., 1999	58	91	45.1 (4.4)	46.7 (3)	ND	No
	França et al., 2009a	48	20	44.8 (8.0)**	49.3 (2.3)	ND	ND
	Suga et al., 2014	18	80	42.7 (3.8)**	47.0 (4.0)	ND	ND
Sensory nerve conduction velocity, sural nerve (m/s)	Klockgether et al., 1999	58	91	44.7 (5.2)	49.0 (4.1)	ND	No
	França et al., 2009a	48	20	45.1 (12.5)**	52.0 (3.0)	ND	ND
	Suga et al., 2014	15	80	47.5 (6.0)	49.6 (4.1)	ND	ND
Motor axon strength-duration time constant	Kanai et al., 2003	20	32	0.48 (0.02)*	0.39 (0.01)	ND	ND

* *p* < 0.05; ** *p* < 0.01; *** *p* < 0.001; # not tested.

BP, Bereitschaftspotential; CES, Cohen effect size; GEEM, gaze-evoked eye movements; ICARS, international cooperative ataxia international rate scale; MEP: Movement-evoked potentials triggered by transcranial magnetic stimulation; ND, not done; NA, no data available; SARA, scale for the assessment and ration of atacia ; SICI: short intracortical inhibition, SRM: standardized response mean.

#### Central neurophysiology

Motor evoked potentials (MEP) evaluate pyramidal tract conductivity by MEP-derived parameters, such as central motor conduction time (CMCT), amplitude, and resting threshold. CMCT in SCA3/MJD was found to be prolonged and associated to clinical scales by some studies (Jhunjhunwala *et al.*, 2013; Farrar *et al.*, 2016). Cortical activity related to movement preparation and execution, and signs of cortical dysfunction in resting motor threshold, short-interval intracortical inhibition (SICI), and cortical silent period duration were found by a recent study, even in presymptomatic SCA3/MJD individuals (Farrar *et al.*, 2016). These markers were strongly correlated to ICARS. Data on SICI and ICARS progression in 18 months were given in mean and standard error of mean. Therefore, CES could not be estimated (Figure 1).

Among sensory evoked potentials, visual evoked potentials (VEPs), brainstem auditory-evoked response (BAER), somatosensory-EPs (SSEPs), pain-related evoked potentials, and sensory gating at hippocampus/brainstem were already studied in SCA3/MJD, and no good candidate has arisen as a state biomarker (Table 2).

#### Video-oculography

Diplopia is a very common finding in patients with SCA3/MJD and can be attributed to ophthalmoplegia or vergence abnormalities. While ophthalmoplegia is easily detected in symptomatic phases of disease, subtle findings such as gaze-evoked and rebound nystagmus, square-wave jerks, saccadic hypermetria, and impaired ocular pursuit are measurable abnormalities described not only in symptomatic (Buttner *et al.*, 1998; Ghasia *et al.*, 2016), but also in presymptomatic carriers (Jacobi *et al.*, 2013; Raposo *et al.*, 2014). Quantitative oculomotor findings have been recently described through video-oculography (Wu *et al.*, 2017). Several parameters were studied, and most of them were shown to be significantly disturbed even in preclinical phases of disease, and to be related to DD and to SARA in later phases (Table 2). A stepwise worsening from pre-ataxic to symptomatic carriers were seen in the frequency and average amplitude of horizontal gaze-evoked eye movements, upward peak saccade velocity, and total antisaccadic error rates. The lowest dispersion rates in pre-ataxic and symptomatic groups were obtained when measuring the upward peak saccade velocity.

#### Vestibular system

Vertigo and imbalance when turning the head are frequent complaints in SCA3/MJD, pointing to involvement of the vestibular system. Measurement of myogenic potentials in the ipsilateral sternocleidomastoid muscle after loud monaural clicks, and of vestibulo-ocular reflex (VOR) after a head impulse test (HIT) were among the neurophysiological evaluations of vestibular dysfunction. VOR disturbances after HIT have been described for a long time in SCA3/MJD (Buttner *et al.*, 1998; Gordon *et al.*, 2003). VOR registrations were improved by using magnetic search coils (Gordon *et al.*, 2014), and video-oculography (VOG) portable systems turned quantitative testing of the VOR possible at the bedside (Agrawal *et al.*, 2014). In a recent study, VOR gain in SCA3/MJD subjects was significantly lower than in controls and correlated with SARA scores in the overall group of ataxic disorders (Luis *et al.*, 2016). VOR dispersion seemed to be larger than SARA dispersion in SCA3/MJD group (Table 2).

#### Peripheral neurophysiology

SCA3/MJD has been associated with axonal neuropathy of both motor and sensory nerve fibers, detected by marked reductions of compound muscle (CMAP) and sensory nerve action potential (SNAP) amplitudes. In addition to sensory losses, muscle cramps might be related to this process, being due to the electrical irritability of unmyelinated nerve twigs, enhanced by collateral sprouting secondary to loss of motoneurons. This electrical irritability of unmyelinated nerve twigs was studied once, and further clarification on this disorder is required (Kanai *et al.*, 2003).

Axonal neuropathy in SCA3/MJD is most probably a neuronopathy rather than a distal axonopathy (Kanai *et al.*, 2003; Escorcio Bezerra *et al.*, 2013), and CMAP and SNAP amplitudes are considered indirect measures of the number of peripheral axons. Axonal neuropathy was mainly explained by age in SCA3/MJD (França *et al.*, 2009a; Klockgether *et al.*, 1999; Linnemann *et al.*, 2016). In a longitudinal observation, sural SNAP showed a significant deterioration after 13 months (França *et al.*, 2009a). The CES of SNAP (0.34) was a little higher than CES of ICARS (0.20) obtained in the same period (França *et al.*, 2009b) (Figure 1).

## Discussion

Several biological fluid compounds and neurophysiological parameters described in SCA3/MJD subjects seemed to be good candidates, but are far from being validated as surrogate state markers for this condition. Most publications described case-control observations where cases were already symptomatic. In contrast, altered results of the peripheral levels of eotaxin and for video-oculography were already found in pre-symptomatic states. Some candidates were associated with disease duration after symptoms onset. The oxidative stress marker GSH-Px, movement-evoked potentials, vestibulo-ocular reflex (VOR), and several video-oculography parameters correlated reasonably and significantly with clinical scales, at this same stage. Only three studies presented a longitudinal design, but no candidate marker was tested in the context of a clinical trial. Validation against a meaningful clinical endpoint was done in some studies. Rate of change in time was obtained for peripheral eotaxin measurements, SICI, and SNAP amplitudes. Although responsiveness to change was not evaluated by the original studies, published parameters permitted us to roughly estimate CES for eotaxin and SNAP. Those values were worse than the ones obtained for the clinical scales (ICARS, SARA and NESSCA) applied simultaneously. It is worth emphasizing that the number of studies that have been designed with the specific aim of identifying biomarkers is extremely limited in this disorder. We could have added other inclusion criteria to our review, such as sample size, existence of technical validation and of a validation cohort, and statistical adjustments in relation to age or gender. Since these additional inclusion criteria would narrow our results, we chose to summarize these and other characteristics in Tables 1 and 2, letting the reader judge about the candidates value for future studies.

SCA3/MJD is a disease essentially confined to the central nervous system. Biological fluid compounds might theoretically reflect the burden of damage related to the disease if they either cross the blood-brain barrier, or are activated both in the CNS and in the periphery. In any case, the search for peripheral compounds is justified by their feasibility in the clinical setting. Although SCA3/MJD pathogenesis is not thoroughly understood and pitfalls might occur in choosing candidates for biomarkers (Aronson, 2005), several clues were already established and are prone to be followed by laboratory studies. Three unbiased surveys aimed to find upregulated genes (Raposo *et al.*, 2015), microRNAs differentially expressed (Shi *et al.*, 2014), and cytokine patterns (da Silva Carvalho *et al.*, 2016) in SCA3/MJD carriers. Preliminary evidence of the first two studies associated overexpression of pro-inflammatory factors FCGR3B and TNFSF14 and the protein encoded by *CLC* to SCA3/MJD, a pattern that subsides with late phases of disease. Furthermore, down-regulation of microRNAs (miR-25 and miR-125b) was associated with activation of astrocytes that got even worse in late phases of the disease. Accuracy and reproducibility have not been established to date for mRNA and miRNA expression analyses, and data were presented as fold change or expression ratios. Moreover, potential superiority of effect sizes cannot be inferred, since dispersion measurements (SE, SEM or SD) and relation to clinical scales were not available.

At least three serum measurements showed interesting characteristics: the already mentioned eotaxin, as well as NSE and GSH-Px (Tort *et al.*, 2005; Zhou *et al.*, 2011; da Silva Carvalho *et al.*, 2016; de Assis *et al.*, 2017). Eotaxin is a peptide secreted not only in peripheral tissues by T-lymphocytes, but also by astrocytes in the CNS (da Silva Carvalho *et al.*, 2016). In the unbiased study on cytokines in SCA3/MJD, eotaxin levels were significantly higher in asymptomatic than in symptomatic carriers or in controls. Although neither correlated to clinical scales nor to disease duration at baseline, eotaxin levels were reduced after 360 days in symptomatic carriers. Eotaxin patterns were in line with results of the microRNA study (Shi *et al.*, 2014), and both unbiased studies raised the hypothesis of astrocyte activation in SCA3/MJD, possibly present in pre-clinical phases, and evolving to exhaustion as the disease progresses. Although eotaxin effect size was small in symptomatic carriers (Figure 1), the effect size in preclinical phases remains unknown. The peripheral indicator of ongoing neuronal damage NSE has been evaluated by two different studies on SCA3/MJD (Tort *et al.*, 2005; Zhou *et al.*, 2011). Increased serum levels of NSE were described by both publications, and the larger study was able to associate NSE to disease duration. In contrast, NSE levels were inversely related to the Extended Disability Status Scale of Kurtzke (EDSS) in the older, and directly related to ICARS and SARA in the more recent study. While this discrepancy remains unsolved, the application of NSE as a potential biomarker is precluded. The activity of the antioxidant enzyme glutathione peroxidase (GSH-Px) was low in SCA3/MJD symptomatic individuals in two studies (Pacheco *et al.*, 2013; de Assis *et al.*, 2017). GSH-Px differences from symptomatic to presymptomatic phases of the disease suggested a temporal association of lower GSH-Px activity to more advanced disease stages, sustaining some expectation in this candidate marker.

Neurophysiological studies have been done based on the hypothesis that the underlying neurological function under study is relevant for SCA3/MJD symptomatology. However, important findings associated to this disease are related to cerebellum and cerebellar-brainstem connections. There is no bedside tool to measure electrophysiological manifestations of cerebellar dysfunction. In spite of that, promising markers emerged from neurophysiology. Among the parameters obtained from MEP, central motor conduction time and SICI were significantly changed and related to ICARS in symptomatic carriers (Figure 1). SICI variability was very large, suggesting that potential CES would be small, for future trials addressed to pyramidal involvement in this disease. VOR is affected in SCA3/MJD symptomatic carriers, and showed a moderate association to SARA, with similar measures of dispersion. Peripheral nerve studies have been performed as well, and sural SNAP showed a significant deterioration after 13 months (França *et al.*, 2009a,b). We were able to estimate CES of both SNAP and ICARS, 0.34 and 0.20, respectively (Figure 1). SARA CES (0.50) was superior to both.

Since they portray brainstem dysfunction, neurophysiological measurements of eye movement abnormalities are very interesting candidate biomarkers. A promising case-control study reported that frequency and amplitude of gaze evoked nystagmus, smooth pursuit eye movements (gain), upward peak velocity and accuracy of saccades, and error rates of antisaccades were already affected in pre-clinical phases of the disease, and were all related to SARA scores and to disease duration in symptomatic carriers (Wu *et al.*, 2016). This results scenario suggests that these manifestations decline in SCA3/MJD in a progressive manner. Although SD of SARA scores was not presented, other observations described SD as being equivalent to 40% to 60% of SARA average results (Jacobi *et al.*, 2011, 2015; Ashizawa *et al.*, 2013; Saute *et al.*, 2014). Some video-oculographic parameters obtained in SCA3/MJD subjects showed proportionally smaller SDs than these figures, like horizontal mean pursuit gain and upward saccadic accuracy (Table 2).

Although evidence levels remain preliminary, the paragraphs below address promising additional biomarkers due to their direct roles in the SCA3/MJD pathophysiology. Molecules associated to quality control systems might play a very relevant role in SCA3/MJD, and we can highlight here two promising ones: beclin-1 and DNAJB1. Beclin-1 is a marker of protein quality control systems, and low protein as well as mRNA levels were found in fibroblasts from symptomatic SCA3/MJD individuals (Nascimento-Ferreira *et al.*, 2011; Onofre *et al.*, 2016). DNAJB1 is a molecular chaperone that stimulates the ATPase activity of Hsp70 heat-shock proteins in order to promote protein folding and prevent misfolded protein aggregation. High DNAJB1 levels were associated with earlier ages at onset than those predicted by the CAG repeat length (Zijlstra *et al.*, 2010). Both compounds should be further evaluated using larger sample sizes and by performing longitudinal observations.

Soluble mutant ataxin-3 levels were measured by time-resolved Forster resonance energy transfer (TR-FRET) immunoassay in human cell lines and brain samples of transgenic SCA3/MJD mice model (Nguyen *et al.*, 2013), but properties of soluble ataxin-3 as a disease biomarker were not addressed up to date. Soluble mutant protein levels have been measured in other neurodegenerative disorders, such as in Huntington disease (HD), and were associated to clinical features (Moscovitch-Lopatin *et al.*, 2013). Soluble huntingtin is currently being evaluated as an outcome in recent HD clinical trials (Huntington Study Group Reach2HD Investigators, 2015; Süssmuth *et al.*, 2015). Likewise measurements of soluble mutant ataxin-3 should be evaluated in future longitudinal studies on SCA3/MJD.

Finally, it is worth to stress that biomarkers are mostly needed for the pre-clinical phases of SCA3/MJD. The pathological process is already on the way before the onset of gait ataxia, and future therapies will probably be more effective if starting early. Studies on pre-symptomatic carriers face more difficulties than others, such as lack of adherence and ethical issues. Fortunately, the time burden measured by the concept “disease duration” since the onset of symptoms and useful for symptomatic studies, can be solved by equations that predict the age at onset and that have recently appeared in the literature (Tezenas du Montcel *et al.*, 2014; Mattos *et al.*, 2019). They will help validating biomarkers for the pre-symptomatic phases.

In conclusion, several potential candidates as state biomarkers have been preliminarily described, albeit through a majority of studies without good sample sizes and/or rigorous designs for the validation of such biomarkers. Candidates for surrogate biomarkers of the pre-symptomatic state were even more scarcely described in the literature. Studies on pre-clinical phases, such as those performed on cytokines and on neurophysiological measurements of eye movement abnormalities, are even more important, since most clinical scales give normal scores in this period. Prospective evaluations are required for all of them, together with measurements of clinical scales and of PGIs. Validation against a MCID, rate of change in time, and responsiveness to change should be established. We are aware that several barriers can delay this goal, including restraints that go beyond the scientists’ efforts and patients’ goodwill. For example, neurophysiology, molecular, and neuroimaging data depend upon technology companies, where planned obsolescence is intrinsic to the production lines. The constant change in platforms turns all knowledge acquisition longer and harder than expected. Hence, solutions for these dilemmas have to be searched for and the future needs to be carefully planned. To this, all-embracing, multi-center studies can be the answer.
